# Retromolar Canal Associated with Age, Side, Sex, Bifid Mandibular Canal, and Accessory Mental Foramen in Panoramic Radiographs of Brazilians

**DOI:** 10.1155/2015/434083

**Published:** 2015-08-20

**Authors:** Ticiana Sidorenko de O. Capote, Marcela de Almeida Gonçalves, Juliana Álvares Duarte Bonini Campos

**Affiliations:** ^1^Department of Morphology, Araraquara School of Dentistry, São Paulo State University (UNESP), Humaita 1680, 14801-903 Araraquara, SP, Brazil; ^2^Department of Social Dentistry, Araraquara School of Dentistry, São Paulo State University (UNESP), Humaita 1680, 14801-903 Araraquara, SP, Brazil

## Abstract

*Background*. The retromolar canal (RMC) is an anatomical variation that can cause complications in dental procedures. *Method*. The RMC was evaluated according to age, sex, and presence of accessory mandibular canal and accessory mental foramen, on both sides in 500 panoramic radiographs, belonging to individuals at the age of 7 to 20 years. The associations of interest were studied through Fisher's Exact Test and Pearson's Chi-Square Test, and the correlation was studied through Pearson's Correlation Coefficient (*r*). The significance level used was 5%. *Results*. The RMC was observed in 44 radiographs (8.8%), and out of those 24 were females. There was no statistically significant association between the RMC and age (*p* > 0.05; Fisher's Exact Test), sex (*p* = 0.787; Pearson's Chi-Square Test), amount of mandibular canals and mental foramina, on both sides (*p* > 0.05; Pearson's Chi-Square Test). There was a significant association between RMC and side, the higher frequency of the canal being on the right side (*p* < 0.05; Fisher's Exact Test). *Conclusions*. Despite the low occurrence of the RMC, its identification and the verification of its dimensions and path are relevant, mainly in cases when anesthetic and surgical procedures can present failures or difficulties.

## 1. Introduction

Difficulties are not rare in the anesthetic practice in lower teeth. Many of them can be related to the variability of the mandibular canal (MC) and its neurovascular content. The retromolar canal (RMC) can be related to those difficulties. It is positioned in the retromolar area of the mandible and it presents morphologic and morphometrical variability [1]. Arch shaped retromolar canals (RMCs) with posterior concavity were observed [2], as well as straight RMCs [3]. Variability in the prevalence of the RMC is also verified in different studies, thus being observed from 1.7% of occurrence of retromolar canals in the area of temporal crest of dry mandibles [4] up to 75.4% of the 171 individuals assessed through tomography exams [5].

The inferior alveolar nerve can cause several extraosseous ramifications before penetrating the mandibular canal, and that variation can be associated with the presence of accessory foramen and multiple canals [6]. A significant correlation between RMC and accessory mandibular foramen (AMF) was observed; however it had no difference between sides or sexes [7].

In the RMCs there may be striated muscle fibers, myelinated nerve fibers, and blood vessels [8]. In the RMC an artery was found, being the branch of the inferior alveolar artery, and the existing nerve derived from the inferior alveolar nerve and went to the third molar region, the retromolar triangle mucosa, the buccal mucosa, the vestibular gingiva of the premolar region, and the inferior molars [9]. Thus, accessory canals in the retromolar region are functionally important in providing the neural and/or vascular components of the mandible [10].

Due to the lack of awareness about the presence of RMCs, an anesthetic failure can happen, and bleeding, paresthesia, or paralysis can occur during odontological procedures. Therefore, despite the little prevalence of the RMC reported in the literature, its identification is interesting.

In dental clinical practice, panoramic radiography is one of the most indicated radiographic examinations by dentists because it provides a general overview of dentomaxillomandibular structures and it is not so costly for patients [11]. Due to the broad coverage of panoramic radiographs, sometimes we can visualize some anatomic variations that are important in dental procedures. In most of the cases, these variations can be identified casually.

The panoramic radiography presents images with less details which may cause difficulties to observe the MC and its variations. Therefore, some authors [12–14] suggest the indication of computed tomography (TC), which allows the observation of three-dimensional structure. From 30 RMCs observed in cone beam computed tomography (CBCT), only 7 were identified in panoramic radiographs [15].

The purpose of this work was to evaluate the prevalence of the RMC and to verify the presence of association according to age, side, sex, and the presence of bifid mandibular canal and accessory mental foramen, on both sides by radiographic evaluation.

## 2. Materials and Methods

The present work was approved by the Committee of Ethics in Research of Araraquara School of Dentistry, Universidade Estadual Paulista (UNESP) (Process number 07/10).

Five hundred (500) panoramic radiographs from individuals at the age of 7 to 20 (average age 13.66 years old) were randomly selected, being 211 males and 289 females. The panoramic radiographs were selected from the records of the Technical Section of Triage, Documentation and Emergency and of the Service of Odontological Radiology of Araraquara School of Dentistry, Universidade Estadual Paulista (UNESP).

The panoramic radiographs utilized were taken with the same equipment (Instrumentarium Imaging Orthopantomograph OP100); the exposure settings were 58–70 kVp/8–10 mA, varying according to the age and biotype. The radiographs were automatically processed in a standardized form (M35-OMAT Processor KODAK), searching to avoid radiographic differences of the retromolar region. Panoramic radiographs were stored under appropriate conditions. Therefore, no alterations related to storage conditions were observed, which might decrease the diagnostic property of the radiographs.

Radiographs with a very radiopaque image, or those which presented metallic structures that made the proper visualization of the region of interest difficult, were not included in the study.

The RMC diagnosis was done by identification of a radiolucent image bounded by radiopaque lines present in the retromolar triangle, associated or not with the distal inferior tooth and/or with MC.

The 500 panoramic radiographs were assessed regarding the presence or absence of the RMC in the mandibular retromolar region on both sides, and the amount of mandibular canals and mental foramina was also observed.

In radiographs where the RMC was visualized, the length, the width (measured from the middle third), the path, and the distance, in relation to the tooth situated in a more distal position in the dental arch, were assessed. The measures were performed with digital calliper (Digit-Cal Plus, Brown & Sharpe).

The radiographs were assessed by an examiner, in a dark room, with a magnifying glass and a negatoscope.

Fifty randomly selected radiographs were assessed by two examiners regarding the presence/number of mandibular canals, mental foramina, and RMC, the length of the RMC, and the width of the RMC on both sides, in order to estimate the interexaminers' agreement. With the purpose of studying the intraexaminer agreement, the fifty radiographs were reassessed, after a one-month period, by the professional who would make the radiographic assessments. Kappa statistics (*k*) and the intraclass correlation coefficient (*p*) were utilized, so respecting the level of mensuration of the variants. The association among the RMC and age, side, sex, and amount of mandibular canals and mental foramina was assessed, on both sides.

The associations of interest (presence/absence of the RMC and age, side, sex, and presence of accessory mandibular canal and accessory mental foramen) were studied through Fisher's Exact Test and Pearson's Chi-Square Test. The correlation between the age, the length, and the width of the RMC was estimated by means of Pearson's Correlation Coefficient (*r*). The significance level used was 5%.

## 3. Results

Regarding the assessment of intraexaminer and interexaminer agreement, all parameters showed an optimal agreement (kappa, *k*), except for the assessment of the right mental foramen which was regular. For the assessments of length and width of the RMC, the agreement was good or excellent (intraclass correlation coefficient, *p*) (Table 1).

In Table 2 we can verify the age according to the number of RMCs, to the absence, and to the unilateral or bilateral presence of the RMC. There was no association between age and presence of the RMC (*p* > 0.05).

**Figure 1:**
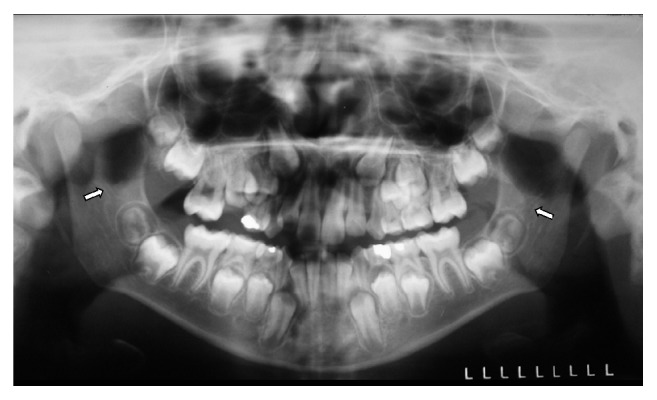
RMC (arrows) observed bilaterally located close to the mandibular third molar germs in a nine-year-old male patient in panoramic radiography.

**Figure 2:**
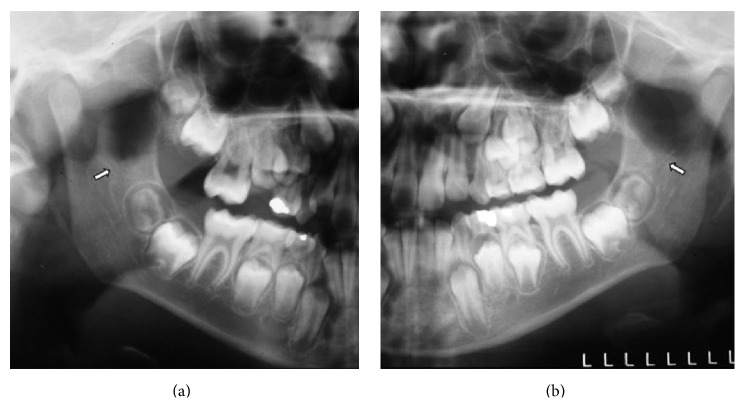
Magnified images of the retromolar regions showing the presence of right and left RMCs (arrows) located close to the right and left mandibular third molar germs, respectively, in a nine-year-old male patient (from Figure 1).

The RMC was observed in 44 radiographs (8.8%), and, out of those, 24 (54.5%) were female. Six men (13.6%) and 4 women (9.1%) presented RMC bilaterally (Figures 1, 2(a), and 2(b) and Tables 3 and 4). Eight men (18.2%) and 11 women (25%) showed one RMC only on the right side, and 6 men (13.6%) and 7 women (15.9%) showed only one RMC (2.3%) on the left side. Two women (4.5%) presented 2 RMCs on the right side (Table 3). Thus, there was no significant association between the presence of the RMC and sex on the right and left sides (Table 4).

In Table 5, the values of length and width of the RMC, on right and left sides, can be observed.

The association between the presence and absence of the RMC and the side was significant, with a higher occurrence of the RMC on the right side (Table 6).

Among the individuals who presented a right RMC (*n* = 31), a nonsignificant correlation was observed between the age variant and its length (*r* = 0.161; *p* = 0.387) and width (*r* = 0.073; *p* = 0.697). Among the individuals who presented a left RMC (*n* = 23), a nonsignificant correlation between the age variant and length (*r* = 0.538; *p* = 0.07) and width of the canal (*r* = 0.083; *p* = 0.700) was also observed.

In Table 7, the amount of mandibular canals and mental foramina, in the assessed individuals, can be observed.

The association between the amount of mandibular canals and the RMC was not significant for the left side, as well as for the right side (*p* = 1.000). Likewise, the association between the presence of RMC and mental foramen was not significant for the left side (*p* = 0.176), as well as for the right side (*p* = 1.000).

The paths of the assessed RMCs were similar, considering that most of them presented one of their extremities (origin) in the foramen region and mandibular canal. After their origin, most of the RMCs presented a curve and descending path, ending in the regions which are close to the teeth that are more distally located in the dental arch.

In 30 individuals, the final extremity of the RMC was in contact with the root of the mandibular third molars, in 5 patients, there was contact with the roots of the inferior second molars, and in 9 individuals, there was no observation of contact with any tooth. Out of those 9, 6 individuals presented one of the extremities of the RMC in the region of the retromolar triangle, considering that the distance from the final extremity of the RMC and the distal face of the third molar ranged from 0.83 mm to 16.46 mm, with an average of 7.47 mm. And in 3 individuals, the RMC ended its path distally to the distal face of the right inferior second molars, with distances of 4.98 mm, 8.66 mm, and 28.14 mm, the last one being located in the mandibular ramus, next to the region of the retromolar triangle, with absence of the third molar in a patient at the age of twenty. However, the two other individuals were at the age of 8 and showed absence of image of the third molar formation.

## 4. Discussion

The mental foramen, the mandibular canal, and their variations as bifid and retromolar canals are anatomic structures considered to be of great clinical interest in surgical and anesthetic procedures.

In the practice of odontological clinic, the panoramic radiograph is one of the diagnostic exams most solicited by the dental surgeons, due to providing a general view of the dentomaxillomandibular structures and to not being so expensive for the patients. Due to those reasons, in this study we assessed RMCs in conventional panoramic radiographs.

In the present study a low occurrence of the RMC (8.8%) was observed. Several studies also assessed the presence of the retromolar foramen and/or canal, so a variation was observed in the prevalence results according to the methodology used (Table 8).

According to some studies, the utilization of the CBCT is important before surgical procedures when there is a suspicion of its evidence in panoramic radiographs [22, 27, 28] and when there is a suspicion of anatomic variation, the computed tomography shows advantages over the panoramic radiograph [23, 29].

The low occurrence found in assessments of the RMCs through panoramic radiographs, when compared to CBCT, is inherent to the radiographic technique itself. Lack of details, irregular magnification, geometric distortion, and overlapping of anatomical structures are the main disadvantages that limit the diagnosis by panoramic radiography [30].

Mandibular canal variations may present false-positive images on panoramic radiographs. This is due to the overlapping of anatomical structures in this type of technique. We can observe a radiological osteocondensation due to the mylohyoid line, which is the insertion area of the mylohyoid muscle and thin cortical outlines due to the imprint of mylohyoid neurovascular bundle in mylohyoid sulcus of the medial surface located on the medial surface of the mandibular ramus. The radiographic image of these structures can be misinterpreted as the mandibular canal variations. A way to differentiate these structures from a bifid mandibular canal image is observing the presence of a characteristic triangular area of bone formed by the cortical outlines of two distinct canals [31].

Another problem related to the panoramic radiograph, which is inherent to the technique, is the appearance of ghost shadows created by the opposing hemimandible, pharyngeal airway, soft palate, and uvula [30] that may cause false-negative images.

Nevertheless, it is important to remember that the panoramic radiograph is the initial radiographic exam for a general evaluation of the patient, and its interpretation can lead to a suspicion of anatomic variations of the mandibular canal. Therefore, when professionals have suspicion of accessory mandibular canals on panoramic radiography, computed tomography should be done to confirm them and avoid complications [11]. CBCT examination has been recommended as a low-cost method with an effective radiation dose less than that of medical CT imaging and only slightly higher than that of panoramic radiography [32, 33]. A clinical study based on three-dimensional images constitutes the only means of providing an irrefutable diagnosis as to the existence of bifid mandibular canals [33], and the detection of the presence of the RMC using CBCT may be crucial for extraction of the mandibular third molars [26].

As well as our results, some other studies found more retromolar foramina on the right side [1, 19, 24]; others did not observe any differences for the right and left sides in dry mandibles [17] or in computed tomography exams [15]. However, in other studies, more RMCs were found on the left side [8, 16, 18, 21].

In the present study there was no association between the presence of the RMC and sex. Studies that assessed the occurrence of RMCs in computed tomography exams or in dry mandibles did not also observe that association [5, 15, 19].

There was no correlation, in our study, between the presence of the RMC on the right and left sides and between the age and length and width of the RMC. Other authors also observed that age did not affect, statistically, the presence of the RMC [15].

In newborns, the mandibular body has little height, its angle is obtuse (130°–160°), the rami are short and wide [34], and the mandibular canal is located near the lower margin [35]. In infant mandible, the body grows in height, with enlargement of the base of body concomitantly with a higher volume of rami, while the angle becomes less obtuse (140°) in the fourth year of life. Over time, the enlargement of the mandible body, distal from the mental foramen, is evident, reducing the angle of the mandible to 120° in adults [34].

The morphological changes that happen over the years in the mandible imply alterations in the positioning of the structures located in the mandibular rami, such as the mandibular foramen, condylar process, and coronoid process, as well as in the mandibular body, such as the path of the mandibular canal and the position of the mental foramen. Likewise, accessory foramina and canals will follow these changes. Therefore, it is important that the pediatric dentists have all this knowledge in order to carry out the necessary adjustments for the anesthetic and surgical procedures.

Regarding the anesthetic procedures, the presence of the RMC may cause anesthetic failures. Since the RMC may contain a neurovascular bundle, the dentist may notice inadequate anesthesia in procedures related to the last tooth in the dental arch or in the retromolar region. Therefore, the solution to this problem would be carrying out other alternative to the conventional inferior nerve technique as Gow-Gates mandibular block technique [36]. The location and configuration of the mandibular canal are important in surgical procedures involving the mandible, such as the extraction of an impacted third molar, dental implant treatment, and sagittal split ramus osteotomy [37].

We observed that the average length and width of the RMC were 12.84 mm and 1.33 mm on the right side and 14.11 mm and 1.49 mm on the left side, respectively. The diameter of the retromolar foramina ranged from 0.5 to 0.2 mm in the study of Bilodi et al. [20].

In the present study, an occurrence of 0.8% of accessory mandibular canals was observed for the right side and 0.2% for the left side, while for accessory mental foramina the occurrence for the right side was 2.8% and 3.2% for the left side. Studies with panoramic radiographs found different values of incidence of bifid mandibular canals: 0.9% in a study with 3612 radiographs [38], 0.08% in 5000 radiographs [39], 0.95% in 6000 radiographs [3], 0.35% in 2012 radiographs [40], and 0.038% in a study with 1000 radiographs [41]. When further studies regarding the bifid mandibular canals were conducted using CBCT, the frequency of bifid mandibular canals increased compared with previous studies using panoramic radiograph [42]. Incidence of bifid mandibular canal using CBCT was 15.6% of 301 hemimandibles [43], 42.8% of 63 patients [24], and 10.2% of 1933 patients [22].

In thirty individuals who presented RMC in this study, the final extremity of RMC was in contact with the root of the mandibular third molars. In another study, the correlation between the presence of the retromolar foramen and the last tooth of the arch was not observed and the distal part of the RMC was projected to the distal root of the third molar and retromolar region. The authors asserted that this distribution shows that the content of that canal innervates and supplies the third molar and the retromolar mucosa of the region [8]. Another study utilizing cadaver mandibles and CBCT verified that the retromolar foramen was located in an average of 14.4 mm posterior from the distal edge of the second molar and 23 mm from the first molar, being located lingually from the mandibular canal [25]. Other authors observed that the retromolar foramen is more commonly found next to the lower half of the temporal crest [17].

Narayana et al. [1] reported that the retromolar foramen can be present in the retromolar triangle and some branches from the inferior alveolar neurovascular bundle would pass through it. Narayana and Prashanthi [44] found one accessory mandibular foramen located posterosuperior to the mandibular foramen. According to the authors, this foramen cannot be considered a retromolar foramen due to its position outside the retromolar triangle and its larger diameter.

## 5. Conclusions

It can be concluded that there was a low occurrence of the RMC in the assessed individuals, causing a significant association regarding side, the higher frequency being on the right side. There were no significant associations regarding age, sex, bifid mandibular canal, and accessory mental foramen.

It is necessary to pay attention to the identification and verification of the dimensions and path of the RMC, mainly in cases when anesthetic and surgical procedures can show failures or difficulties. When there is any suspicion of alteration in the mandibular canal, we suggest the indication of more accurate complementary exams as the cone beam computed tomography.

## Figures and Tables

**Table 1:** Intraexaminer and interexaminer agreement.

Measure^*∗*^	Examiner 1	Examiner 1 × 2
	*k*	*k*
MC(R)	1.00	1.00
MC(L)	1.00	1.00
MF(R)	0.55	0.47
MF(L)	0.90	0.81
RMC(R)	0.81	0.94
RMC(L)	0.93	0.93

	*ρ* (IC_95%_)	*ρ* (IC_95%_)

RMCL(R)	0.72 (0.50–0.84)	0.88 (0.79–0.93)
RMCL(L)	0.93 (0.88–0.96)	0.89 (0.81–0.94)
RMCW(R)	0.76 (0.58–0.86)	0.80 (0.65–0.89)
RMCW(L)	0.91 (0.85–0.95)	0.84 (0.72–0.91)

^*∗*^MC(R): right mandibular canal, MC(L): left mandibular canal, MF(R): right mental foramen, MF(L): left mental foramen, RMC(R): right retromolar canal, RMC(L): left retromolar canal, RMCL(R): length of the right retromolar canal, RMCL(L): length of the left retromolar canal, RMCW(R): width of the right retromolar canal, and RMCW(L): width of the left retromolar canal.

**Table 2:** Descriptive statistics of the age variant according to the number of retromolar canals on both sides.

Retromolar canal	*n*	%	Age			
			Minimum	Maximum	Average	Standard deviation
Right side						
No canal	469	93.8	7	20	13.77	4.66
1 canal	29	5.8	7	20	12.28	4.82
2 canals	2	0.4	8	10	9.00	1.41
Left side						
No canal	477	95.4	7	20	13.77	4.67
1 canal	23	4.6	7	20	11.50	4.30
Retromolar canal						
Absent	458	91.6	7	20	13.83	4.66
Unilateral	32	6.4	7	20	12.30	4.57
Bilateral	10	2.0	7	20	10.92	4.40

**Table 3:** Descriptive statistics of sex variant according to the number of retromolar canals on both sides.

	Sex				Total	
RMC	Male		Female			
	*n*	%	*n*	%	*n*	%
Right						
Absent	197	39.4	272	54.4	469	93.8
1 canal	8	1.6	11	2.2	19	3.8
2 canals	—	—	2	0.4	2	0.4
Bilateral	6	1.2	4	0.8	10	2
Total	**211**	**42.2**	**289**	**57.8**	**500**	**100**
Left						
Absent	199	39.8	278	55.6	477	95.4
1 canal	6	1.2	7	1.4	13	2.6
Bilateral	6	1.2	4	0.8	10	2
Total	**211**	**42.2**	**289**	**57.8**	**500**	**100**

**Table 4:** Study of association between the retromolar canal (absent, unilateral, and bilateral) and sex.

	Sex				Total		
RMC	Male		Female				*p* ^*∗*^
	*n*	%	*n*	%	*n*	%	
Absent	191	38.2	265	53	456	91.2	
Unilateral	14	2.8	20	4	34	6.8	0.787
Bilateral	6	1.2	4	0.8	10	2	

Total	211	42.2	289	57.8	500	100	

^*∗*^Pearson's Chi-Square Test.

**Table 5:** Values of length and width of the retromolar canal, on the right and left sides, measured in millimeters.

RMC	Length (mm)			Width (mm)		
	Average	Minimum	Maximum	Average	Minimum	Maximum
Right side	12.84	6.74	22.27	1.33	0.52	2.74
Left side	14.11	4.99	23.25	1.49	0.30	2.79

**Table 6:** Study of association between presence and absence of the retromolar canal and the side.

	RMC(L)				Total		
RMC(R)	Absent		Present				*p* ^*∗*^
	*n*	%	*n*	%	*n*	%	
Absent	456	91.2	13	2.6	469	93.8	0.000
Present	21	4.2	10	2	31	6.2	

Total	477	95.4	23	4.6	500	100	

^*∗*^Fisher's Exact Test.

**Table 7:** Amount of mandibular canal and mental foramen in the assessed individuals (*n*).

Variant	*n*	%
MC(R)		
1 canal	496	99.2
2 canals	4	0.8
MC(L)		
1 canal	499	99.8
2 canals	1	0.2
MF(R)		
1 foramen	486	97.2
2 foramina	14	2.8
MF(L)		
1 foramen	484	96.8
2 foramina	16	3.2

Total	500	100.0

**Table 8:** Other studies in the literature with regard to sample characteristics and incidence of occurrence of retromolar canals (RMC) or retromolar foramen (RMF).

Studies	Year	Sample size	Region of population	Sample type	Sample age (years)	Incidence of RMF/RMC
Schejtman et al. [16]	1967	18	Argentine	Human heads' cadavers	—	72% RMC

Athavale et al. [17]	2013	71/10	India	Dry mandible/adult cadavers	—	14.08% RMF in dry mandibles/0% RMF in adult cadavers

Narayana et al. [1]	2002	242	India	Dry adult mandibles	—	21.9% RMF

Bilecenoglu and Tuncer [8]	2006	40	Turkey	Mandibles	—	25% RMF

Rossi et al. [18]	2012	222	Brazil	Dry adult mandibles	—	26.58% RMF

Galdámes et al. [19]	2008	294	Brazil	Dry adult mandibles	18–100	12.9% RMF/RMC

Bilodi et al. [20]	2013	41	India	Dry mandible	—	12.19% RMF

Gupta et al. [21]	2013	50	India	Dry adult mandibles	—	18% RMF

Kang et al. [22]	2014	1933	Korea	CBCT images of patients	16–57	5.38% RMC

Patil et al. [5]	2013	171 patients (254 images)	Japan	CBCT images of patients	15–79	75.43% RMC

Lizio et al. [23]	2013	187 patients (233 images)	Italy	CBCT images of patients	Mean age of 46	16% RMC

Orhan et al. [24]	2013	63 patients (126 images)	Turkey	CBCT images of patients (children)	7–16	22.20% RMC

Kawai et al. [25]	2012	46 patients (99 images)	Japan	CBCT images of cadaver mandibles	—	52% RMF

von Arx et al. [15]	2011	100 patients (121 images)	Switzerland	CBCT images of patients and panoramic radiographs	16–83	25.6% (CBCT) and 5.8% (panoramic radiographs) RMC

Sisman et al. [26]	2015	632 patients (947 images)	Turkey	CBCT images of patients and panoramic radiographs	15–58	26.7% (CBCT) and 3.06% (panoramic radiographs) RMC
